# Genomic and Clinical Correlates of Adrenocortical Carcinoma in an Adult Patient with Li-Fraumeni Syndrome: A Case Report

**DOI:** 10.3390/curroncol28010025

**Published:** 2020-12-31

**Authors:** Suraya Bondy, Camilla Tajzler, Sebastien J. Hotte, Anil Kapoor, Kevin Zbuk, Aly-Khan A. Lalani

**Affiliations:** 1 Faculty of Health Sciences, McMaster University, Hamilton, ON L8S 4L8, Canada; bondys@mcmaster.ca; 2 Division of Urology, Department of Surgery, McMaster University, Hamilton, ON L8S 4L8, Canada; tajzlec@mcmaster.ca (C.T.); akapoor@mcmaster.ca (A.K.); 3 Division of Medical Oncology, Department of Oncology, McMaster University, Hamilton, ON L8V 5C2, Canada; hotte@hhsc.ca (S.J.H.); zbuk@hhsc.ca (K.Z.)

**Keywords:** Li-Fraumeni Syndrome, adrenocortical carcinoma, next-generation sequencing

## Abstract

Li-Fraumeni Syndrome (LFS) is defined by germline mutations of the p53 tumour suppressor gene. Adrenocortical carcinoma (ACC) is a rare aggressive malignancy that is commonly associated with LFS. Most LFS-linked ACC cases occur in children, and limited research has been dedicated to the clinical outcomes and genomics of adult cases with LFS-linked ACC. We report on a 34-year-old female who was diagnosed with three separate malignancies: stage III invasive ductal carcinoma of the right breast, metastatic ACC from the right adrenal gland, and grade 2 pleomorphic sarcoma of the left hand. Her invasive breast ductal carcinoma was treated with neoadjuvant chemotherapy, and she received a bilateral mastectomy after her LFS was confirmed with genetic blood testing. Adrenal ACC was initially treated with a right nephrectomy and adrenalectomy, followed by adjuvant mitotane and two lines of chemotherapy after disease recurrence. Her hand sarcoma was treated by second ray amputation. Further, we conducted deep next-generation sequencing of each of her unique tumour tissue samples using FoundationONE CDx. A whole-genome shot capture followed by in vitro sequencing performed by the Illumina^®^ HiSeq platform revealed a germline P191fs*18 TP53 mutation across all three tissue samples. This case provides insight into the genomics and clinical characteristics of LFS-linked adult-onset ACC and demonstrated that p53 mutations were preserved throughout each malignancy, without apparent treatment pressures on genomic profiling. This case reinforces the critical importance of adopting best practices for LFS, which include the implementation of highly vigilant screening and management of care in a multidisciplinary setting.

## 1. Introduction

Li-Fraumeni Syndrome (LFS) is a rare cancer predisposition syndrome defined by autosomal dominant germline mutations of the TP53 tumour suppressor gene [1,2]. A diagnosis of LFS confers a lifetime cancer risk of ≥70% in males and ≥90% in females [2]. It is associated with malignancies including soft tissue sarcomas, osteosarcomas, central nervous system tumors, breast cancers, and adrenocortical carcinomas (ACC) [2].

ACC is recognized as a core LFS-related cancer, comprising 12% of all tumours associated with the disorder and more commonly seen in children [3]. While childhood cases of ACC are highly associated with germline TP53 mutations, this association is less clear in adults [3]. The majority of genomic interrogation has been explored in either childhood cases with ACC or adults with sporadic ACC; however, less study has been conducted on a per-patient level in adult ACC cases with LFS [4]. Herein, we report on a case of LFS displaying adult-onset ACC and provide clinical and genomic annotation of her three separate malignancies.

## 2. Case Description

**Figure 1:**
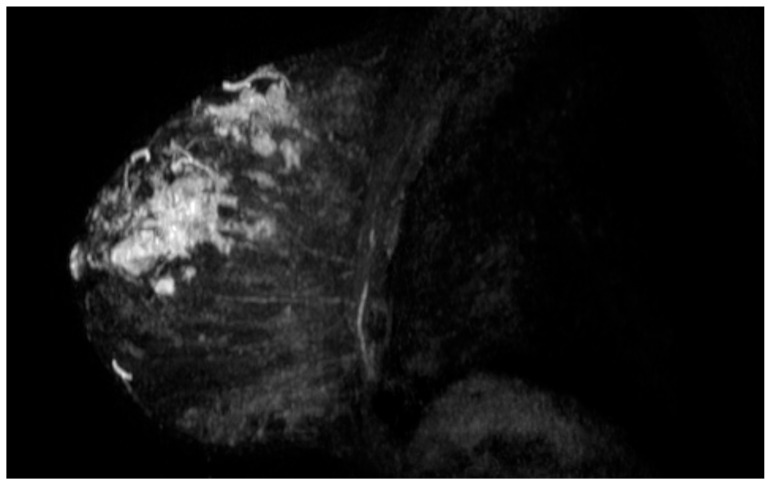
Magnetic resonance imaging revealing the 10 cm ductal carcinoma in the right breast.

A 33-year-old female presented to Juravinski Cancer Centre at McMaster University with a large palpable mass on the right breast and lymphadenopathy in the right axilla. Relevant family history included her mother deceased at age 45 from glioblastoma, and her maternal grandmother deceased at age 20 from a possible sarcoma. Ultrasound guided biopsy revealed a grade III, lymph-node positive invasive ductal carcinoma, 10 cm, noted to be estrogen receptor/progesterone receptor (ER/PR) negative and human epidermal growth factor receptor 2 (HER2) positive (Figure 1). One month later, the patient underwent genetic testing and was diagnosed with LFS. A 19 gene panel identified a pathogenic mutation in the TP53 gene. The patient received neoadjuvant chemotherapy with 4 cycles of doxorubicin plus cyclophosphamide and 4 cycles of paclitaxel. She then underwent a bilateral mastectomy and was treated adjuvantly with radiation to the right chest wall and 13 cycles of Herceptin. There was no sign of residual malignancy post-surgery.

**Figure 2:**
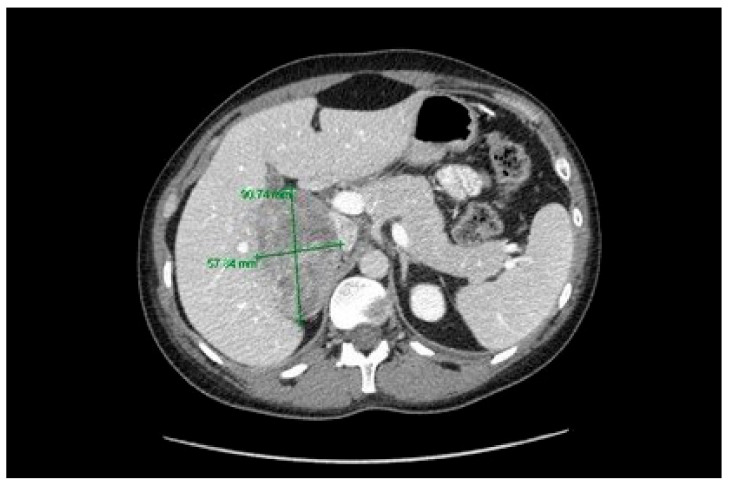
Computed tomography revealing the 9.1 × 5.8 cm right adrenocortical mass.

After 9 months on Herceptin, the patient presented with fatigue, hypertension, and a cushingoid appearance. Laboratory testing revealed an elevated cortisol level of 811 nmol/L (normal range: 150–550 nmol/L). A computed tomography (CT) scan revealed a right adrenal mass measuring 9.1 × 5.8 cm (Figure 2). The patient underwent an open radical right adrenalectomy and nephrectomy with positive margins. Post-operative pathology reported a high-grade pT2 pNX ACC with unremarkable kidney architecture. The patient elected to receive adjuvant mitotane, commencing at 2 g daily.

**Figure 3:**
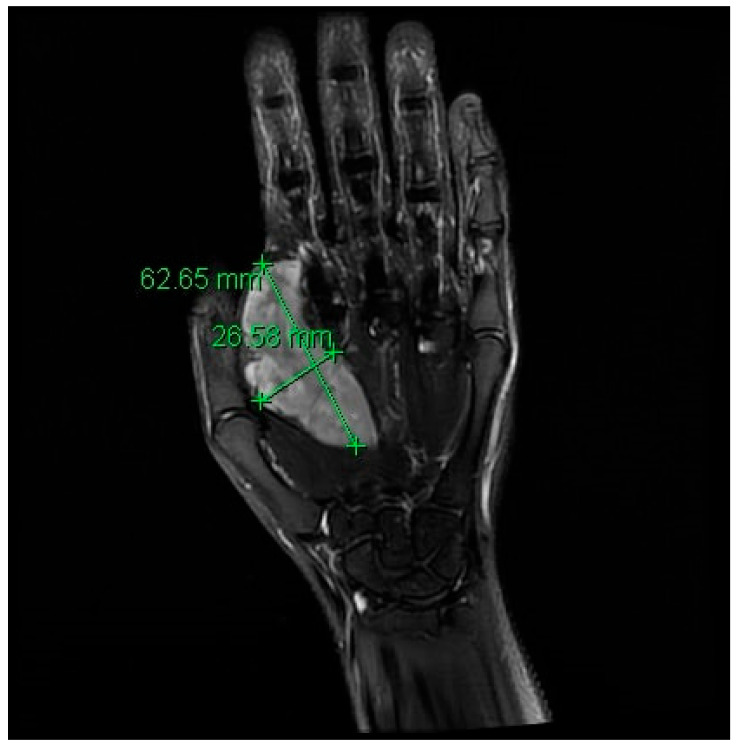
Magnetic resonance imaging revealing the 6.3 × 2.7 cm soft tissue sarcoma of the left hand.

During surveillance follow-up, a CT scan 1-year post-surgery revealed a peritoneal nodule measuring 2.9 cm, a significant mass involving the intrahepatic inferior vena cava (IVC) and nodular lesions along the adrenalectomy site and retroperitoneum. Biopsy confirmed the peritoneal nodule as metastatic ACC (mACC). The patient was treated with 6 cycles of palliative cisplatin and etoposide chemotherapy and continued on mitotane daily. After treatment, CT showed overall stable disease in lymphadenopathy and peritoneal nodules. Concurrently, the patient noted a rapidly enlarging mass along the radial border of her second ray index finger, causing weakness and decreased range of motion. Magnetic resonance imaging (MRI) and biopsy confirmed the mass as grade 2 undifferentiated pleomorphic sarcoma (Figure 3). She underwent a left hand second ray amputation with no post-operative complications. Four months later, surveillance imaging showed an increase in gastrohepatic lymphadenopathy with mass effect on the IVC. The patient received second-line gemcitabine and capecitabine chemotherapy with mitotane up-titrated to 4 g daily. After a good response at 4 months, she was assessed for potential surgical resection at the National Institutes of Health. However, she was not a surgical candidate due to complexity and unavailability of vascular surgery there. A CT scan two months later (off of therapy) revealed disease progression in the right upper quadrant mass, periportal mass, and the right flank with a new lesion in the gastrohepatic region. She then restarted gemcitabine and capecitabine, which she continues on currently.

With patient consent and in line with institutional approvals (Hamilton Integrated Research Ethics Board, Project 5175-T, approved 2 December 2019), next-generation sequencing was performed on the patient’s three histological samples from (a) right mastectomy, (b) right adrenalectomy, and (c) left hand second ray amputation. Formalin-fixed paraffin-embedded tissue from all three tumours was used for genomic testing of 324 cancer-related genes through FoundationONE CDx, as described previously [5]. Results indicated a preserved TP53 mutation across all tumour types, as well as an ERBB2 amplification in the breast sample and an alteration of the MEN1 gene in the adrenal sample (Table 1). Tissue results were negative for BRCA1 and BRCA2, low tumour mutation burden, and microsatellite stable. Efforts are now being made to secure treatment with CDK4/6 inhibitors based on the noted MEN1 mutation (Table 1).

## 3. Discussion

ACC is a rare aggressive cancer affecting 1–2/million people [6]. Typical clinical presentation features are often secondary to excessive glucocorticoid and androgen secretion. Data along the disease spectrum suggest the median overall survival is approximately 14.5 months [7]. Results from Ayala-Ramirez et al. (*n* = 330) suggest that tumour size, older age, functioning tumours, disease stage, and incomplete resection are associated with poor prognosis [6]. Currently, detection at ENSAT stage I-II with radical tumour resection provides a potential for cure in patients with ACC. Postoperative adjuvant mitotane therapy is recommended for at least 2 years based on international guidelines [8,9]. Treatment for advanced ACC builds upon mitotane monotherapy, including etoposide, doxorubicin, and cisplatin; streptozotocin; and/or gemcitabine plus capecitabine, depending on patient response [8]. However, prognosis remains poor [8]. Various clinical trials are investigating options to expand the therapeutic armament, including cabazitaxel (NCT03257891), cabozantinib (NCT03612232), combination camrelizumab and apatinib (NCT04318730), combination nivolumab and ipilimumab (NCT03333616), and pembrolizumab (NCT02673333).

With respect to predictive or prognostic biomarkers, PD-L1 expression has garnered interest in ACC given the current permeation of immunotherapy in genitourinary treatment paradigms. While one report suggests that PD-L1 positivity can be as high as 70% of patients, there was no significant association between PD-L1 expression and adverse clinico-pathologic features or outcomes (*n* = 28) [10]. Genetically, ACC has frequently been correlated with germline TP53 mutations in childhood cases [4]. The median age of onset for ACC is 4.8 years among germline TP53 mutation carriers versus 41.9 years among sporadic ACC cases [11]. Adult ACC cases appear to demonstrate a lower frequency of germline TP53 mutations. Hermann et al. found that only 3.9% of adult ACC patients display a germline TP53 mutation (*n* = 103), which is significantly less than what is shown in childhood data (50–80%) [4]. Ultimately, the genomic characterization of adult ACC cases is complex with several different oncogenic alterations having been shown to effect disease prognosis [12,13]. Interestingly, 20–55% of predominantly autosomal MEN1 mutations have been associated with adrenal lesions and also noted in a small number of adult cases of ACC [14].

Our case builds upon and is consistent with previous clinical and genomic characterization of patients with ACC in relation to LFS. We captured granular clinical information and genomic characterization in a young patient with three separate malignancies. Using next generation sequencing, a TP53 frameshift insertion mutation was identified within exon 6 across all three tumour types, without apparent treatment pressures on genomic profiling [15]. Integrating genomics into this case revealed the biological underpinnings of disease and has provided a basis to seek CDK4/6 inhibitors as another line of therapy with a unique mechanism of action [16]. CDK4/6 inhibitors have promising early results in smaller preclinical and clinical study for ACC and rarer sarcomas [16,17]. Methods of managing of LFS are complex due to relative rarity; variation in gene expression; associated cancer types; and evolving practice on cancer surveillance, prevention, and treatment [1]. This case reflects the importance of advocating for a spectrum of diagnostic and therapeutic measures for young patients with rare tumours. Given the guarded outcomes in these clinical scenarios, LFS patients may benefit from interventions ranging from routine screening to genomic sequencing and clinical trials. Furthermore, this case underscores the importance of managing patients in a multidisciplinary setting to ensure cohesive care plans.

## 4. Conclusions

We present the genomic and clinical characteristics of a patient with LFS-linked adult-onset ACC, demonstrating that TP53 mutations were preserved throughout each malignancy without apparent treatment pressures on genomic profiling. This case reinforces the importance of establishing best practices for LFS, which include detailed screening, interrogation of potential driver mutations, and developing care plans in a multidisciplinary setting.

## Figures and Tables

**Table 1:** FoundationONE CDx results of next generation sequencing results from respective tumour tissue samples.

Tissue Sample	ERBB2	FGFR4	NOTCH3	BRCA1	BRCA2	MEN1	TP53
Breast invasive ductal carcinoma	amplification	amplification	amplification	negative	negative		P191fs*18
Adrenocortical Carcinoma						Splice site 840—2A>T	P191fs*18
Soft Tissue Sarcoma							P191fs*18

Abbreviations: ERBB2, erb-b2 receptor tyrosine kinase 2; FGFR3, fibroblast growth factor receptor 3; NOTCH3, notch 3; BRCA1, breast cancer gene 1; BRCA2, breast cancer gene 2; MEN1, multiple endocrine neoplasia type 1; TP53, tumor protein p53.
